# Combining multiple predictive markers in stratified medicine

**DOI:** 10.1186/1745-6215-16-S2-O85

**Published:** 2015-11-16

**Authors:** Richard Emsley, Matthias Pierce, Graham Dunn

**Affiliations:** 1 Centre for Biostatistics, Institute of Population Health, The University of Manchester, Manchester, UK; 2 Manchester Academic Health Science Centre, Manchester, UK; 3 MRC North West Hub for Trials Methodology Research, Manchester, UK

## 

Stratified medicine requires identifying predictive markers to determine differential treatment effects for subgroups. Ideally, these markers are then validated in prospective randomised trials. However, many of the methods for the analysis and validation of markers are based on there being a single marker. For example, a biomarker stratified trial design typically stratifies on a single binary marker. In many clinical scenarios, including recently funded MRC consortia, there are hypothesised to be multiple markers, often of different modalities such as clinical, genetic and imaging markers.

One issue in identifying any predictive marker is that an interaction effect is additive to the main effects of both treatment and the marker, which are also included in the analysis model. With multiple markers, if the effect size of one marker is much larger than any others, it alone may be sufficient for stratification of the population. If not, it may be appropriate to combine all markers into one single predictive biomarker.

Kraemer has proposed a method for combining multiple markers into a single combined measure, provided the markers are not highly correlated, which is likely when they share a common mechanism. We describe this approach, and extend it to account for multiple markers of different modalities and which are identified from different data sources. We will discuss the issue of choosing the appropriate scale of interaction (additive versus multiplicative), and how to incorporate multiple markers into prospective trial designs such as biomarker stratified designs.

